# Organic-inorganic materials containing nanoparticles of zirconium hydrophosphate for baromembrane separation

**DOI:** 10.1186/s11671-015-0758-x

**Published:** 2015-02-12

**Authors:** Yuliya S Dzyazko, Ludmila M Rozhdestvenskaya, Yu G Zmievskii, Alexander I Vilenskii, Valerii G Myronchuk, Ludmila V Kornienko, Sergey V Vasilyuk, Nikolay N Tsyba

**Affiliations:** Department of Sorption and Membrane Materials and Processes, V.I. Vernadskii Institute of General and Inorganic Chemistry, NASU, Palladin Pr. 32/34, 03142 Kiev, Ukraine; Department of Process Equipment and Computer Technology Design, National University of Food Technologies of the Ministry of Education and Science of Ukraine, Vladimirskaya str. 48, 01601 Kiev, Ukraine; Department of Membrane Technologies, A.V. Shubnikov Institute of Crystallography, RAS, Leninskii pr. 59, Moscow, 119333 Russian Federation; Department of Carbon Sorbents for Medical and Ecological Application, Institute for Sorption and Problems of Endoecology, NASU, General Naumov Str. 13, 03163 Kiev, Ukraine

**Keywords:** Organic-inorganic membranes, Active layer, Zirconium hydrophosphate, Nanoparticles, Baromembrane separation

## Abstract

Organic-inorganic membranes were obtained by stepwise modification of poly(ethyleneterephthalate) track membrane with nanoparticles of zirconium hydrophosphate. The modifier was inserted inside pores of the polymer, a size of which is 0.33 μm. Inner active layer was formed by this manner. Evolution of morphology and functional properties of the membranes were investigated using methods of porosimetry, potentiometry and electron microscopy. The nanoparticles (4 to 10 nm) were found to form aggregates, which block pores of the polymer. Pores between the aggregates (4 to 8 nm) as well as considerable surface charge density provide significant transport numbers of counter ions (up to 0.86 for Na^+^). The materials were applied to baromembrane separation of corn distillery. It was found that precipitate is formed mainly inside the pores of the pristine membrane. In the case of the organic-inorganic material, the deposition occurs onto the outer surface and can be removed by mechanical way. Location of the active layer inside membranes protects it against damage.

## Background

Application of ultrafiltration involves a wide variety of fields, for instance, recovery of ionic species (usually enhanced by polyelectrolytes) [1,2], treatment of brackish [3] and waste water [4], food industry (for juice concentration [5], protein recovery from whey [6]) and medicine [7]. Both polymer and ceramic membranes are used for baromembrane processes [8].

Almost all the commercially available membranes contain thin nanoporous active layer applied to macroporous substrate. A thickness of the active layer is up to several micrometers. The active layer is necessary to provide separation ability of the membranes, and the macroporous substrate guarantees their low hydrodynamic resistance. In the case of polymer membranes, the active layer is formed, particularly by interfacial polycondensation, plasma polymerization, *in situ* polymerization at the outer surface of the membrane, polymer grafting [9]. Sol-gel method is often applied to the formation of active layer of inorganic membranes [10].

In opposite to fragile inorganic materials, polymer separators are more attractive for operation due to their elasticity and stability of small pores, which determine permittivity of the membranes, against high pressure. However, foiling of the membranes by organic species as well as development of microorganism debris inside the polymer decreases a lifetime of the membranes on the one hand and declines permeate fluxes on the other hand [11,12]. In order to minimize fouling with organics and microorganisms, insertion of nanoparticles of inorganic compounds, such as SiO_2_ [13,14], particularly stabilized with N-halamine [14], ZrO_2_ [15], Fe_2_O_3_ stabilized with chitosan [16] and TiO_2_ [17], into polymers has been proposed. Two approaches were used for the preparation of ultrafiltration [13-16] and reverse osmotic membranes: insertion of sol or suspension containing the inorganic constituent into the dissolved polymer or vice versa [13-16] as well as modification of the polymer membrane, which had been prepared preliminary [17]. These approaches require further coupling of the obtained film with macroporous substrate or use of polymer composite membranes consisting of the substrate and active layer. Another problem is a purposeful formation of needed porosity. In the case of modification of preliminary formed polymer membrane, a question of necessity of multiple modification is still opened.

Moreover, fouling of the membranes requires their periodical cleaning, which is often carried out mechanically or by means of hydrodynamic pulsation [11,12]. This causes damages of thin active layer and, as a result, shortage of their lifetime. At last, complex and expensive equipment is needed for industrial manufacture of the composite membranes.

Earlier electrodialysis membranes were obtained by formation of active layer inside macroporose ceramics. ZrO_2_ nanoparticles were found to block macropores of the membrane and form secondary porosity [18]. Pores between these particles as well as high surface charge density provide semipermittivity of the membranes towards anions in acidic media and towards cations in alkaline solutions [19,20]. This gives a possibility to assume a similar approach to create polymer-based organic-inorganic membranes also for ultrafiltration.

In this work, the membranes were tested by deionized water and corn distillery. In the last case, the ultrafiltration allows us to remove useful components (crude proteins, fat etc.) [21], which can be further used for preparation of livestock feed. Simultaneously, ecological problem of wastewater purification can be solved.

## Experimental

### Track membranes

Track membrane has been chosen as a model polymer matrix since its porous structure involves through regular pores, a size of which is several hundreds nanometers [22]. Studies were performed using a poly (ethyleneterephthalate) (PETP), a thickness of which was 11 μm. Preliminary, the film was irradiated with Xe ions with an energy of 1 MeV/nucleon and a density of 2 × 10^9^ ions cm^−2^ under the vacuum environment of 10^−6^ Torr similar to [23,24]. The energy of incident ions was sufficient to form through latent tracks. Then, UV sensibilisation was carried out for restructurisation of fragments of molecular compounds in order to shorten the period of subsequent etching. The etching was performed in a KOH solution (250 mol m^−3^) at 348 K.

### Modification of the polymer matrix

Polymer matrix was filled with zirconium hydrophosphate (ZHP), a choice of the modifier due to its chemical stability and possibility to obtain nanosized particles inside polymer pores [25-28]. In opposite to hydrated zirconium dioxide, which was used for the modification of ceramics, ZHP is characterized by higher surface charge density in neutral solutions. In prospect, the membranes can be used for other tasks, which require this property. Moreover, a treatment of the immersed polymer with an alkaline solution for deposition of hydrated zirconium dioxide can result in damage of the membrane material.

Sol of insoluble zirconium hydroxocomplexes was prepared and analysed as described earlier [18]. The membrane was boiled in deionized water under vacuum, treated with a H_3_PO_4_ solution (1,000 mol m^−3^), dried at ≈ 298 K and heated at 343 K, the ion-exchanger was removed from the outer surface of the membrane by means of ultrasonic activation at 30 kHz using a Bandelin device (Bandelin Electronic GmbH & Co. KG, Berlin, Germany). Then, the membranes were dried at 343 K down to constant mass, weighted and stored in a desiccator over CaCl_2_. The modification was repeated several times, after each modification cycle a sample was taken for investigations.

### Morphology and porosity of the membranes

Both outer surface and transverse section of the membranes were investigated using a JEOL JSM-6060 LV scanning electron microscope (JEOL Ltd., Akishima-shi, Japan), elementary analysis of the modifier incorporated into the polymer was provided by this manner. Preliminarily, the samples were coated with an ultrathin gold layer at 3 Pa by means of an auto fine coater JEOL JFC-1600 (JEOL Ltd.).

A fine-dispersed powder was obtained from the composite by its grinding under cooling with liquid nitrogen. The powder was researched using a JEOL JEM 1230 transmission electron microscope (JEOL Ltd.).

Micro- and mesopores were determined by means of nitrogen desorption using a Quantachrome Autosorb 6B analyzer (Quantachrome instruments, Boynton Beach, FL, USA). Before the measurements, the samples were vacuumized at 343 K. Bulk density *ρ*_*b*_ was estimated from mass and geometrical sizes of the membrane, particle density was found with a picnometer (Archimedes) method similar to [29]. Total porosity (*ε*) was calculated as $1-\frac{p_b}{p_p}1-\frac{p_b}{p_p}$.

### Ion-exchange capacity and membrane potential

Cation-exchange capacity of the membranes was determined by their multiple treatment with a NaCl solution (100 mol m^−3^), washing with deionized water (electrical conductivity of the effluent was performed), treatment with a HCl solution (100 mol m^−3^) and analysis of the effluent using a PFM-U flame photometer.

Membrane potential was measured at 298 K using a two-compartment divided cell similar to [30,31]. Pairs of NaCl solutions (0.05 to 5 and 10 mol m^−3^) filled their chambers, where Ag/AgCl electrodes were placed.

### Separation process

**Figure 1:**
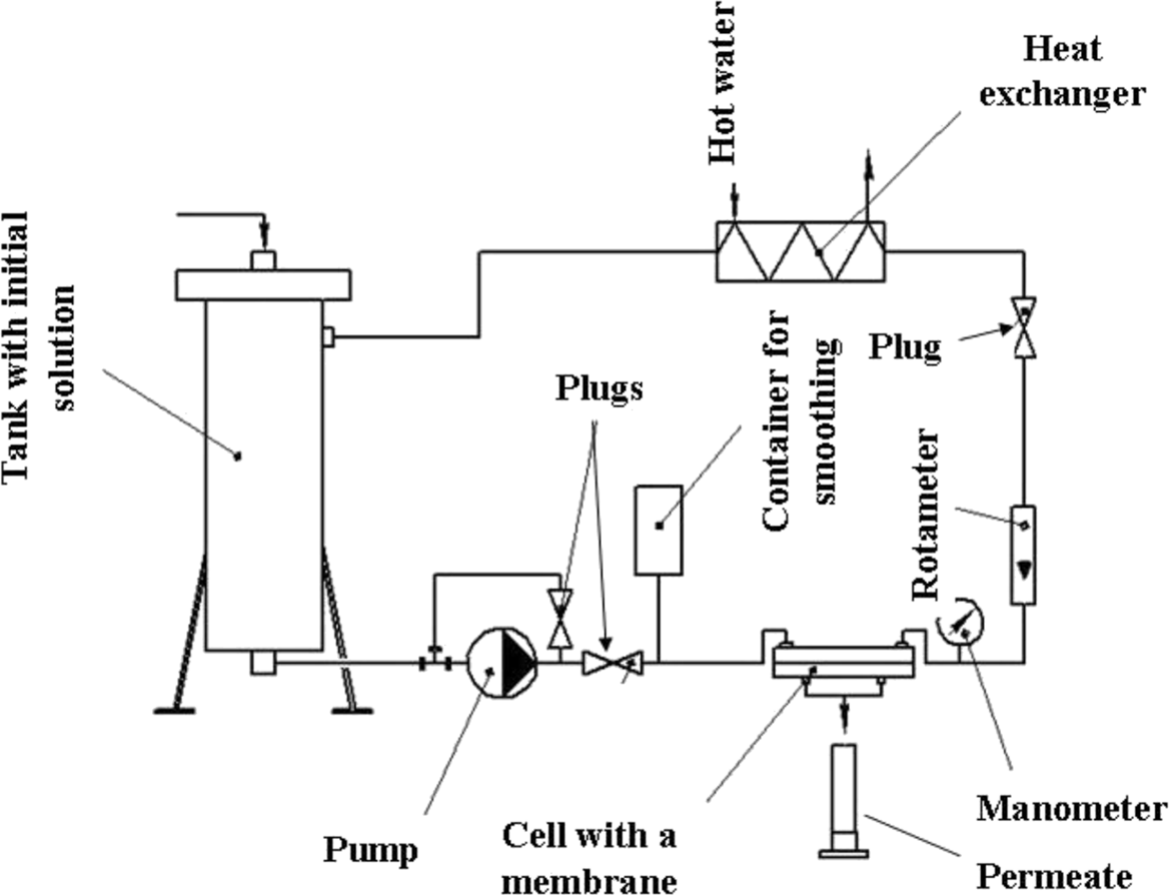
**Experimental set-up for baromembrane processes.**

Experimental set-up involved a plane membrane cell, liquid line, thermostat and pressure and flow controllers (Figure 1). The effective membrane area was 2.1 × 10^−3^ m^2^.

Preliminary testing was as follows. Initially deionized water was passed through the membrane at 0.3 MPa and 333 K for 16 h. After the crimping by this manner, the membrane was stored at room temperature and atmosphere pressure for 24 h. Then, the passage was continued in order to determine the resistance of the membrane. After this, water was replaced by corn distillery, which had been preliminary centrifuged and filtered using Buchner funnel. The separation was performed for 4 h (the pressure was kept at 0.1 or 0.3 MPa), then the liquid was replaced by deionized water to find the membrane resistance again. After this, the membrane was removed from the cell, cleaned, dried and investigated with SEM and porosimetry methods. A content of the matters in the permeate and concentrate was determined with a refractory method.

The membranes (both the pristine one and just after modification) were also tested several times. First of all, the crimping was performed as described above. The separation cycle was as follows. Corn distillery was passed through the system at 0.3 MPa for 4 h. When the separation process was finished, the membrane was removed from the cell, its outer surface was cleaned mechanically and washed with deionized water. The membrane was stored in aqueous medium for 20 h. After this, the membrane was inserted into the cell again and tested with deionized water. The separation cycles were repeated five times. Then, the membrane was stored in deionized water for 96 h, washed with a 0.1 M HCl solution and water up to neutral reaction of the effluent. The separation cycle was carried out again.

## Results and discussion

### Morphology and porosity of the pristine membranes

**Figure 2:**
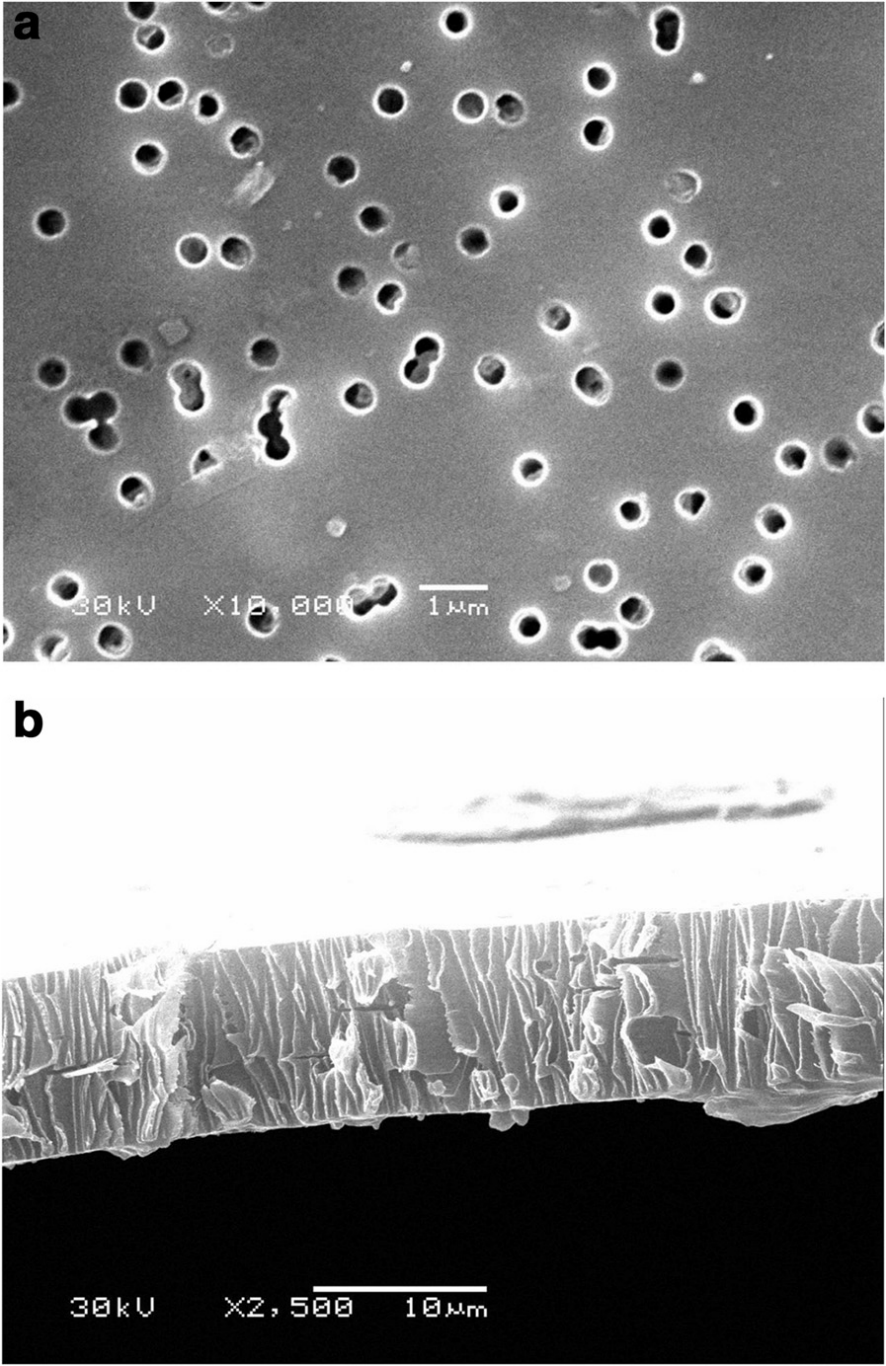
**SEM image of outer surface (a) and cross-section (b) of the pristine membrane.** Through pores, a shape of which can be assumed as cylindric, are visible.

SEM images of surface and cross-section of the pristine track membrane are represented in Figure 2. Round holes of regular shape are seen at the surface, the size of the holes is 0.33 μm. In general, no roughness is visible around the circumference of the holes indicating evidently smooth walls of the pores. A distance between holes is up to several micrometers. Some holes are double and even triple. Though pores are seen in the SEM image of a cross-section, some pores show tortuosity (however, most of them are straight), some of them merge and branch. Assuming cylindrical and regular shape of the pores, the porosity has been estimated as 0.1 by means of analysis of ten images. This in an agreement with data obtained with a picnometer method (Table 1).

**Table 1:** **Characteristics of the membranes**

***m***	***ε***	**Volume of micropores, cm** ^**3**^ **g** ^**−1**^	***S*** **, m** ^**2**^ **g** ^**−1**^	***A × *** **10** ^**3**^ **, mmol g** ^**−1**^	***η*** **, C m** ^**−2**^	***r*** **, nm**
0	0.109	7.01 × 10^−5^	1.9	5.9	0.030	158
0.047	0.082	2.32 × 10^−4^	8.2	1.5	0.018	4.8
0.052	0.080	3.35 × 10^−4^	11.8	2.3	0.019	3.1
0.056	0.075	3.86 × 10^−4^	13.6	2.8	0.020	2.2
0.061	0.070	4.57 × 10^−4^	16.1	3.3	0.020	3.2
0.063	0.066	5.06 × 10^−4^	17.9	3.8	0.021	2.7

**Figure 3:**
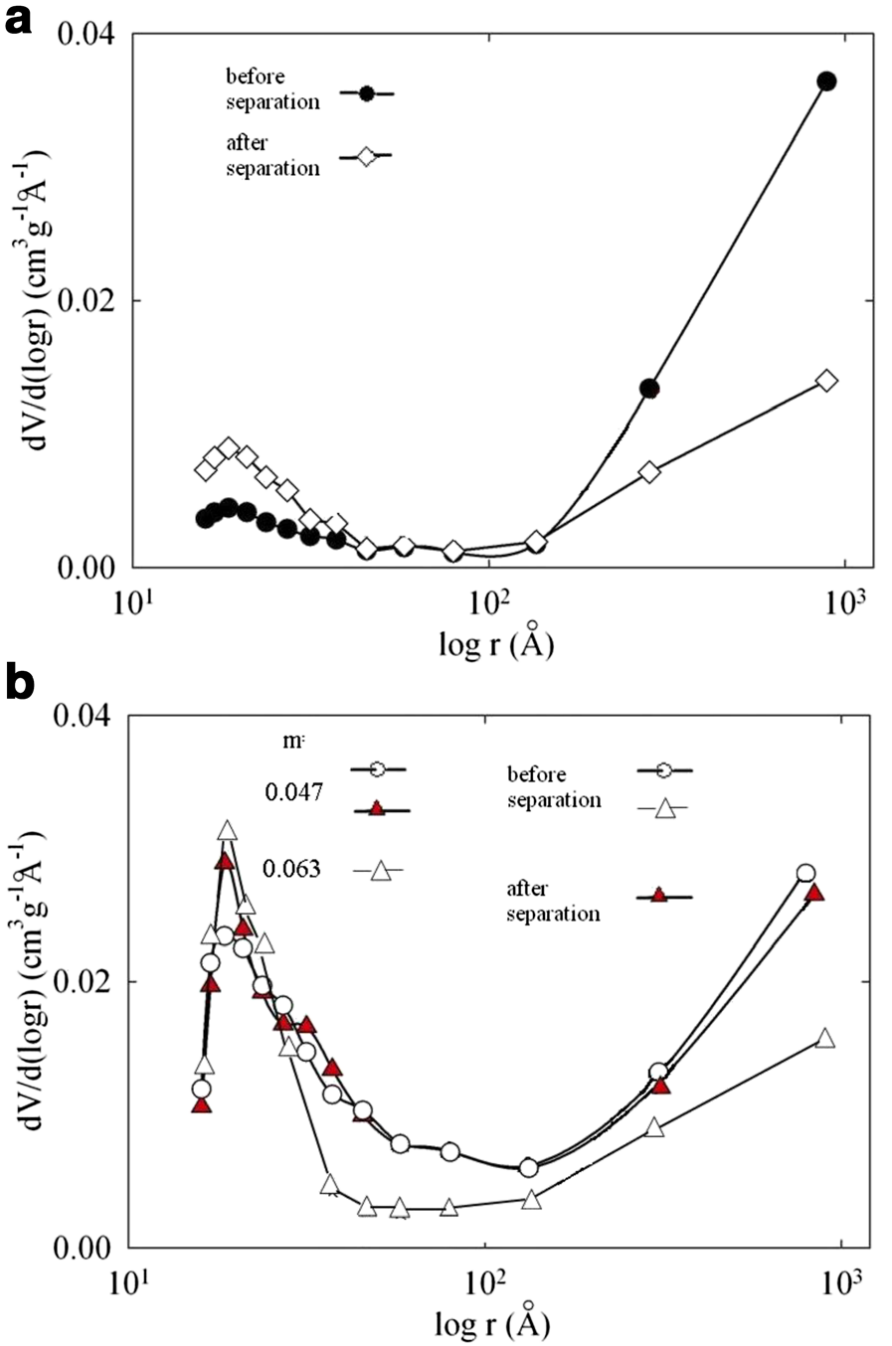
**Differential pore size distributions.** Obtained for pristine polymer **(a**) and organic-inorganic membrane containing 4.7 and 6.3 mass % ZHP **(b)**. The pristine **(a)** and composite (4.7% ZHP) **(b)** membranes were investigated before and after the separation process.

Differential pore size distribution is given in Figure 3. Two peaks are visible: the first one as well as micropores corresponds to pore radius (*r*) up to 4.5 nm and evidently related to polymer heterogeneities. The second peak is attributed through pores, they are partially outside the region of sensitivity of the method. Wide peak is evidently due to tortuosity and merger-branching of the pores.

### Morphology and porosity of the modified membranes

As shown earlier with methods of dynamic laser light scattering and TEM, sol of insoluble zirconium hydroxocomplexes includes both single globular nanoparticles, a minimal size of which is 4 nm, and their aggregates [18]. The particles with a diameter of 15 (non-aggregated globules) and 120 nm dominate in sol. Pores of the pristine membrane are available both for nanoparticles and their aggregates.

Stepwise modification, which involves removal of the precipitate from outer surface of the membranes, results in an increase of ZHP content inside the polymer (see Table 1). The largest growth of mass fraction (*m*) of the inorganic constituent is reached during the first modification cycle. Smaller increase of the *m* value is achieved during further modification, no sufficient growth of ZHP amount has been found after the fifth cycle. In owing to this, no further modification was performed.

**Figure 4:**
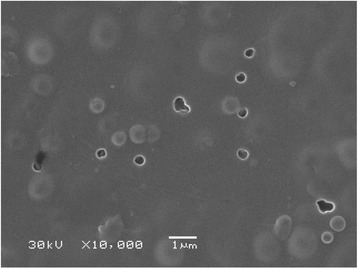
**CEM image of the organic-inorganic membrane.** Pores attributed to the polymer are visible only partially.

A major part of holes becomes invisible in SEM image of the outer surface of the modified sample (Figure 4). Some round convexities are seen. Larger size and larger distance between them than for the pristine membrane indicate blocking and stretching of the macropores and their partial squeezing from the side of filled pores.

**Figure 5:**
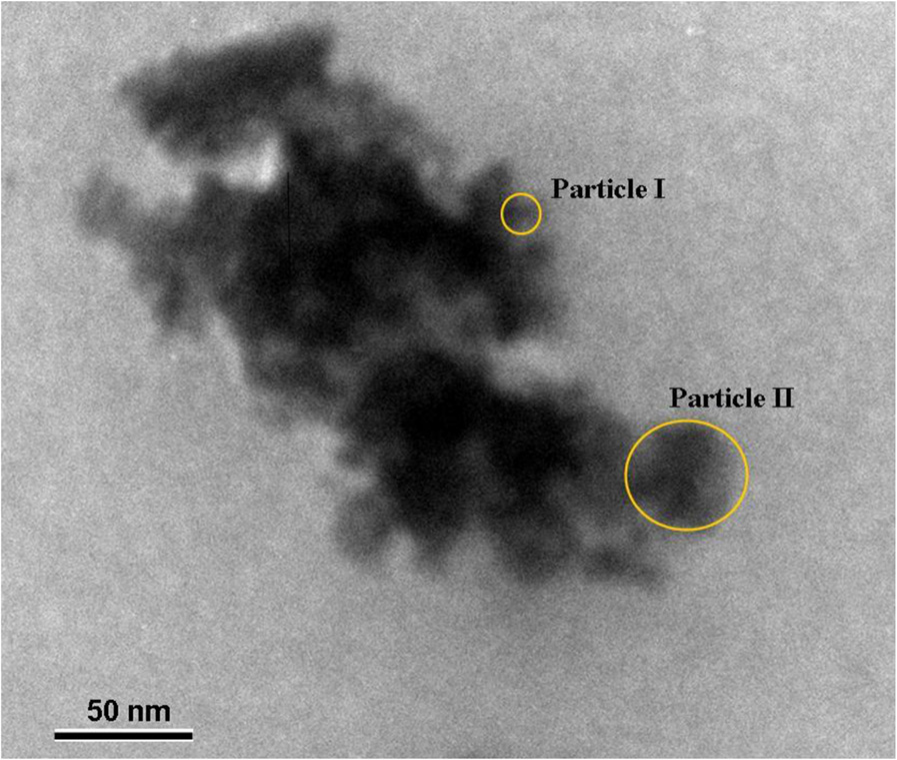
**TEM image of ZHP agglomerate incorporated into the polymer.** The nanoparticles, which form aggregates, are visible.

As follows from Table 1, increasing of ZHP amount in the polymer results in a growth of microporosity and specific surface area (*S*), the total porosity decreases simultaneously. Differential pore size distribution shows a higher peak at *r* = 1.7 nm in a comparison with that of the pristine membrane. Moreover, the second narrow peak at *r* = 3 nm is visible for the membrane with a minimal ZHP content. This peak indicates a presence of larger particle than those which form smaller pores. Indeed, TEM image of the membrane powder shows the agglomerate, which consists of aggregates, a size of which is from 30 nm (Figure 5). The aggregates include smaller nanoparticles. The peak at *r* = 3 nm practically disappears for the membrane with a maximal content of the modifier.

Regarding to the pristine membrane, its cation-exchange capacity (*A*, see Table 1) is caused by − COOH groups, which are formed during etching of the polymer by alkaline solution [23]. Insertion of ZHP into the polymer predictably causes increases of capacity. It should be noted, that a Zr:P molar ratio was ≈ 1:1.9 for all the samples, this is rather close to that for crystalline material (α-ZHP modification [32]). Moreover, the membranes demonstrate an increase of exchange capacity with increasing of the modifier amount.

### Incorporated modifier

**Figure 6:**
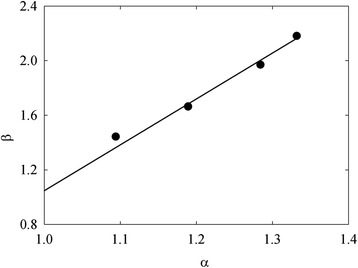
***β*** **parameter as a function of α parameter.**

Microporosity of the membranes is undoubtedly attributed to the modifier. In order to estimate loosening-compactness of the porous structure of the modifier on the level of micropores, the *α* and *β* parameters have been proposed. The *α* parameter is a $\frac{m_n}{m_1}$ ratio, where the ‘1’ index corresponds to the one-time modified membrane (i.e. to minimal ZHP content), ‘*n*’ is related to membranes containing larger ZHP amount. Similarly, the *β* parameter corresponds to a $\frac{V_{micr,n}}{V_{micr,1}}$ ratio, where *V*_micr_ is a volume of micropores. Regarding the membrane with a minimal content of the modifier, *α* = *β* = 1. In our case, the *β* − *α* plot is linear (Figure 6). Since $\frac{d\beta }{d\alpha }$ > 1 ($\frac{d\beta }{d\alpha }$ = 3.3), stepwise modification causes loosening of porous structure of the filler due to deposition of more friable microporous formations from cycle to cycle.

**Figure 7:**
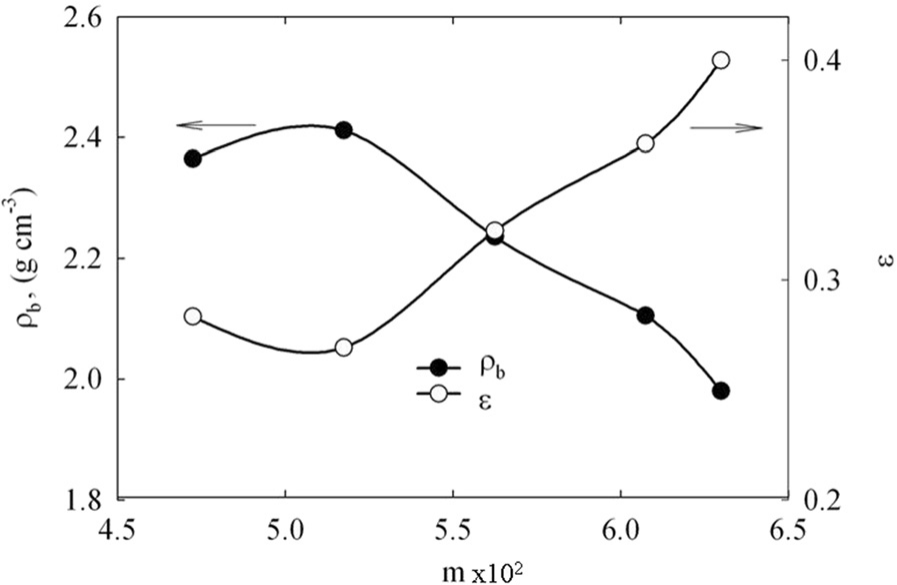
**Bulk density of the inner ZHP layer and its porosity.** These characteristics are given as functions of mass fraction of the modifier.

In a framework of the first approximation, bulk density of the incorporated modifier (${\rho}_b^{/}$) can be determined from the *m* value and decrease of porosity. The porosity of ZHP (*ε*^/^) was calculated as $1 - \frac{\rho_b^{/}}{\rho_p^{/}}$, where ${\rho}_p^{/}$ is the particle density (3.3 g cm^−3^ for crystalline α-ZHP modification [32]). The plots of ${\rho}_p^{/}$ − *m* and *ε*^/^ − *m* demonstrate the maximum and minimum, respectively (Figure 7), which is evidently a result of a contradiction of two reasons: increase of microporosity on the one hand and decrease of mesopore volume on the other hand.

**Figure 8:**
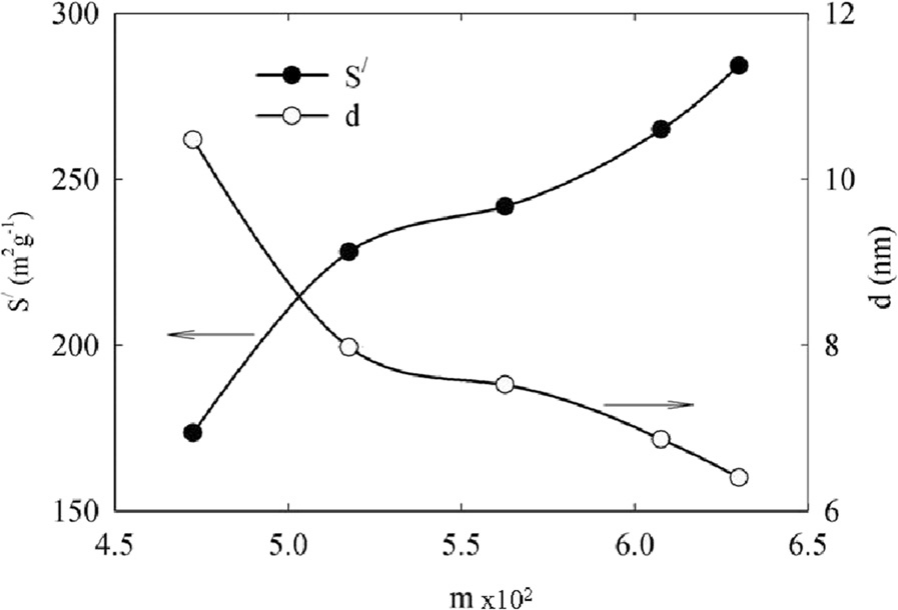
**Specific surface area of the modifier and particle diameter.** These characteristics are given as functions of mass fraction of the modifier.

Specific surface area of incorporated ZHP demonstrates a growth with increasing of the modifier content evidently due to development of microporosity (Figure 8). Diameter of the globules was calculated as $\overline{d}=\frac{6}{\rho_p^{/}{S}^{/}}$ [33]. As seen from the figure, effective diameter of the particles decreases with increase of the *m* value indicating deposition of the smallest particles inside the membranes from stage to stage of the modification.

### Potentiometric transport numbers

In all the cases, the membrane potential was registered. Regarding the pristine membrane, it contains − COOH groups, which are formed during etching of the polymer with alkaline solution. These groups are dissociated partially in neutral media. An excess of counter ions (Na^+^) in the diffusion parts of electric double layer causes its slightly expressed charge-selective properties towards cations. In the case of organic-inorganic membranes, the membrane potential is due to the dissociation of (−О)_2_РО_2_Н and –0РО_3_Н_2_ groups:

1 $$ {\left(-\mathrm{O}\right)}_2{\mathrm{PO}}_2\mathrm{H}\to {\left(-\mathrm{O}\right)}_2{{\mathrm{PO}}_2}^{-}+{\mathrm{H}}^{+} $$

2 $$ \hbox{--} 0{\mathrm{PO}}_3{\mathrm{H}}_2\to \hbox{--} 0{\mathrm{PO}}_3{\mathrm{H}}^{-}+{\mathrm{H}}^{+} $$

3 $$ \hbox{--} 0{\mathrm{PO}}_3{\mathrm{H}}^{-}\to \hbox{--} 0{{\mathrm{PO}}_3}^{2-} + {\mathrm{H}}^{+} $$

**Figure 9:**
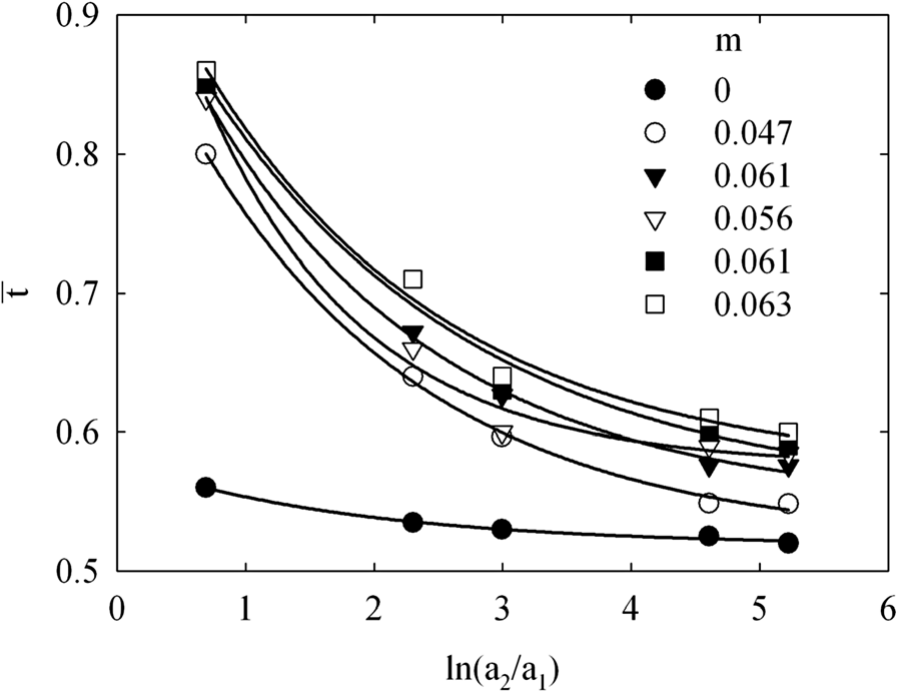
**Transport number of counter-ions as a function of** $\boldsymbol{n}\frac{{\boldsymbol{a}}_{\boldsymbol{2}}}{{\boldsymbol{a}}_{\boldsymbol{1}}}$ **.**

In the last case, the transport number of counter-ions ($\overline{t}$) through the membrane was determined from the data of membrane potential (*E*_*m*_) according to the formula for 1,1 binary electrolyte [31]:

4 $$ {E}_m=\frac{RT}{F}\left[ \ln \frac{a_2}{a_1}\pm 2{\displaystyle \underset{a_1}{\overset{a_2}{\int }}\left(1-\overline{t}\right)d \ln {a}_{\pm }}\right] $$

where *a*_1_ and *a*_2_ are the activities of counter-ions in less and more concentrated solutions, respectively, *a*_±_ is the activity of the solution of varied concentration (more concentrated solution in our case), *R* is the gas constant, *F* is the Faraday constant and *T* is the temperature. The transport numbers of Na^+^ ions are represented in Figure 9, they are sensitive to the solution concentration and approximated to the ‘true’ value with a decrease of a difference of the solution concentration [30]. This value is evidently realized under applied potential.

A radius of pores, which provide the membrane potential, can be calculated from the expression [34]:

5 $$ \overline{t}=t\left(1+\frac{F\overline{r}C}{k\overline{\eta}}\right){\left(t+\frac{F\overline{r}C}{k\overline{\eta}}\right)}^{-1} $$

where *t* is the transport number of counter-ions (Na^+^) in a solution (0.4), *k* is the shape coefficient (*k =* 2.8 for pores formed with globules), *η* is the surface charge density and *C* is the solution concentration. The surface charge density (see Table 1) was found as $\frac{FA}{S}$.

**Figure 10:**
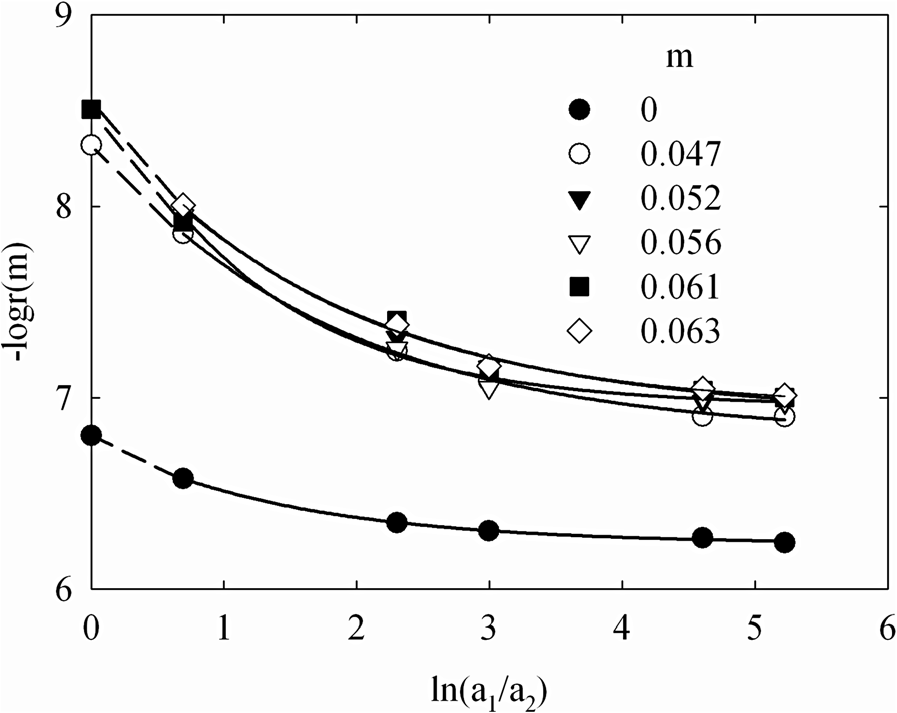
**Logarithm of pore radius as a function of** $\boldsymbol{ln}\frac{{\boldsymbol{a}}_{\boldsymbol{2}}}{{\boldsymbol{a}}_{\boldsymbol{1}}}$ **.** Calculations were performed according to Equation 5.

Equation 5 gives the transport number, at which the concentrations of the solutions from two sides of the membranes are close to each other. In other words, extrapolation of the curve like *r* − $ln\frac{{\mathit{\mathsf{a}}}_{\mathit{\mathsf{2}}}}{{\mathit{\mathsf{a}}}_{\mathit{\mathsf{1}}}}$ to the ordinate axis allows us to obtain ‘true’ *r* magnitude (Figure 10). These data are shown in Table 1. The result obtained for the pristine membrane is in a good agreement with SEM observation. However, in the case of modified membranes, the potentiometric method gives nanosized values, which are in a contradiction with porosimetric measurements (they show a presence of larger pores).

**Table 2:** **Hydrodynamic resistance of the membrane and their selectivity (preliminary testing)**

	**Pretreatment with water,** ***R*** **, m** ^**−1**^		**Separation,** ***ϕ*** **, %**		**Treatment with water after separation,** ***R*** **, m** ^**−1**^	
***m***	***τ*** **= 0**	***τ*** **= 16 h**	***ΔP*** **= 0.1 MPa**	***ΔP*** **= 0.3 MPa**	***ΔP*** **= 0.1 MPa**	***ΔP*** **= 0.3 MPa**
0	0.11 × 10^13^	1.81 × 10^13^	31	31	4.35 × 10^13^	5.25 × 10^13^
0.047	0.81 × 10^13^	2.13 × 10^13^	31	34	4.85 × 10^13^	5.75 × 10^13^

**Figure 11:**
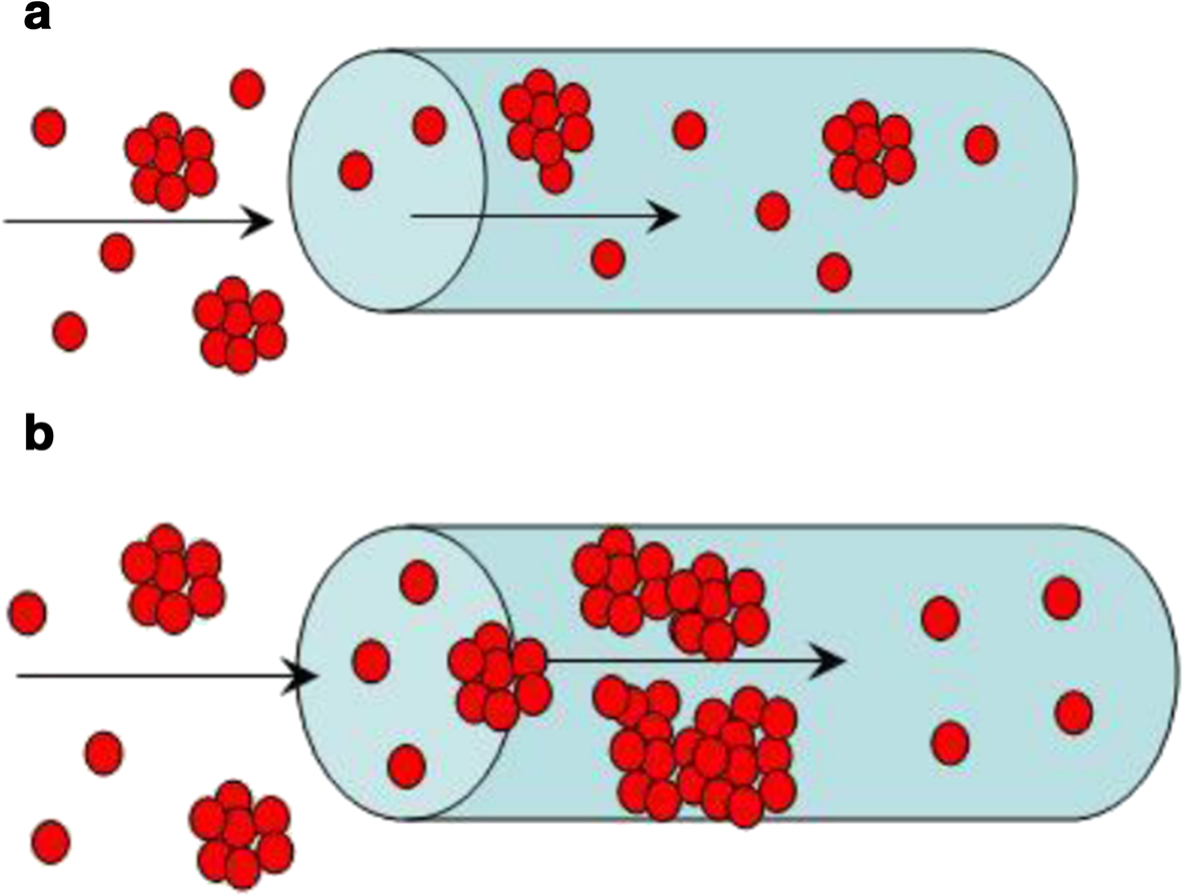
**Filling of the membrane pores during the first (a) and further (b) stages of modification.** The nanoparticles inside the polymer macropores block them and form secondary porosity.

Thus, a mechanism of filling of the polymer matrix with pores, which are smaller than 1 μm, is similar to those for ceramics (*r >* 1 μm). Matrix pores are blocked with aggregates of ZHP nanoparticles during the first synthesis stage (Figure 11). The aggregates evidently give pores, a radius of which is about 4 nm (see Figure 4). The aggregates isolate wide cavities, which are partially seen in the differential pore size distributions. During further modification stages, only nanosized particles of sol are able to penetrate inside matrix pores. Pores between the aggregates are gradually blocked with ZHP nanoparticles, making full filling of macropores of the polymer impossible. Since the modifier occupies about 30% of the total pore volume, its maximal thickness is ≈ 3 μm (assuming that all the modifier form ‘corks’).

### Baromembrane separation

In order to characterize the membrane behaviour during the process, the experimental data were analyzed as follows. A flux (*J*) of permeate was determined as [8]:

6 $$ J=\frac{3,600V}{A\tau } $$

Here, *V* is the permeate volume, *A* is the effective membrane area and *τ* is the time. Selectivity of the membranes (φ) was estimated according to the expression:

7 $$ \varphi =\left(1-\frac{C_2}{C_1}\right)\times 100\% $$

where *C*_1_ and *C*_2_ are the concentration of species in concentrate and permeate, respectively. At last, hydrodynamic resistance (*R*) of the membrane was calculated according to Darcy equation:

8 $$ J=\frac{\varDelta P}{\mu R} $$

where *μ* is the dynamic viscosity and *ΔP* is the pressure drop.

**Figure 12:**
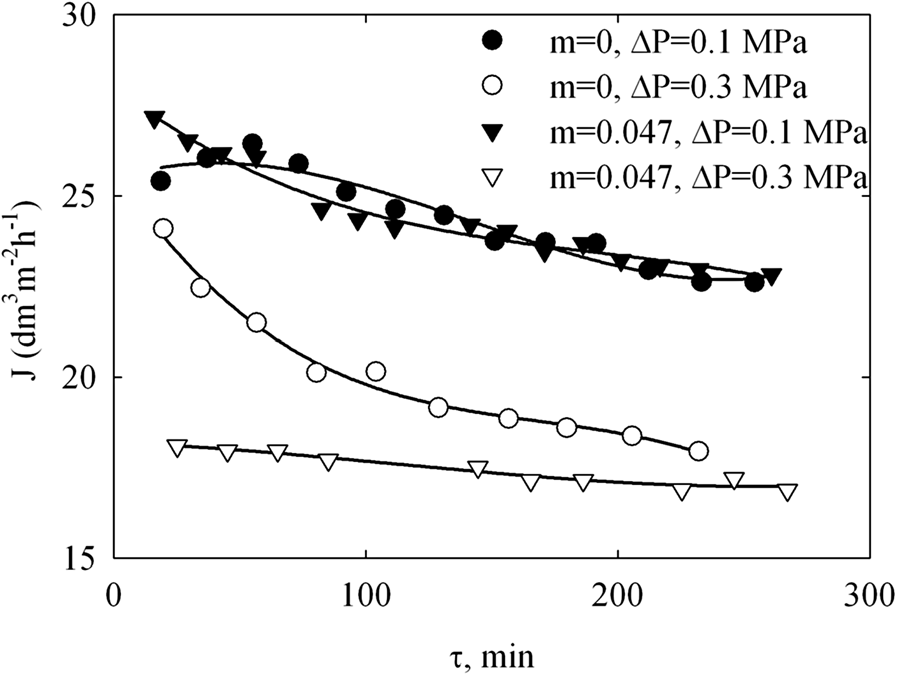
**Flux of permeate through pristine and modified membranes as a function of time.**

Hydrodynamic resistance towards water is predictably higher for the membrane containing ZHP (*m* = 0.047) than that for the pristine membrane (Table 2). Other materials showed the resistance, which was higher in two times in a comparison with the pristine separator, these materials were not used for testing. As seen from the table, a flux of water through the membrane tends to increase with a growth of pressure drop. In a contrary, a flux of the permeate obtained during separation of corn distillery decreases with increasing of pressure (Figure 12). Moreover, the *J* value decreases in time due to fouling. The most stable flux has been found for the modified membrane at 0.3 MPa. In other cases, the flux gradually decreased in time. No sufficient difference of selectivity was found for the modified membrane in a comparison with the pristine separator (see Table 2). It means less size of species in the distillery (<8 nm) than pore size of the membrane. These species are able to penetrate through the membrane into permeate.

**Figure 13:**
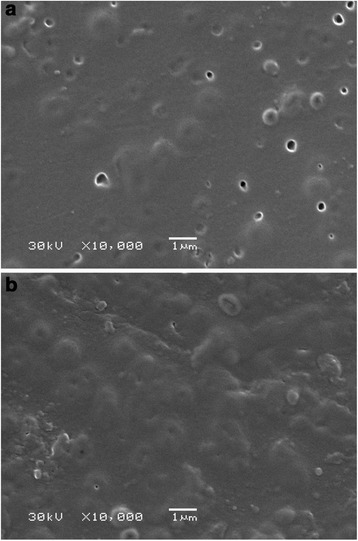
**Morphology of the modified (a) and pristine (b) membranes after separation.**

After the end of the processes, higher amount of precipitate was found on the outer surface of the modified membrane, the membrane shows higher hydrodynamic resistance. No sufficient difference of pore size distribution was found for the modified membrane (see Figure 3). In opposite to composite material, the pristine membrane demonstrates considerable increase of porosity due to pores with *r* < 4.5 nm. This growth is evidently caused by particles of organics, which are deposited inside pores. At the same time, a volume of through pores is considerably lower in comparison with that for the membrane, which is free from a precipitate. Moreover, a change of morphology of the polymer membrane is seen in the SEM image (Figure 13, compare with Figure 2). On the other hand, the images of the modified membrane before and after the process are practically the same.

Thus, in the case of the pristine membrane, a decrease of the flux is evidently caused by the precipitate formation inside the membrane. Regarding the modified separator, the fouling is evidently due to deposition onto outer surface. This precipitate can be easy removed by mechanical way or by hydrodynamic pulsation, thus, the organic-inorganic membrane looks more attractive from the practical point of view. Location of the active layer inside the membrane prevents its damage.

The data obtained for repeated testing are given in Table 3. In the case of pristine membrane, hydrodynamic pressure increases dramatically just after the first operation cycle. Cleaning of the membrane did not provide removal of the precipitate from pores. No considerable growth of this parameter was found for the modified membrane during the next operation cycles. Further change of the resistance is within the statistical error indicating fouling mainly during the first cycle. The modified membrane demonstrates lower resistance. If no removal of the precipitate from outer surface of the separator was provided, a ratio of resistances of the modified and pristine membrane is 1.2 (see Table 2). As follows from Table 3, this ratio becomes 0.5 after cleaning. It means the modified membrane accumulates organics only onto outer surface. Regarding the pristine membrane, both outer surface and pores are poisoned.

**Table 3:** **Hydrodynamic resistance of the membrane and their selectivity (repeated testing at 0.3 MPa)**

**Cycle number**	**Pristine membrane**		**Modified membrane (** ***m = *** **0.047)**	
	**Selectivity,** φ**, %**	**Treatment with water after separation and cleaning,** ***R*** **, m** ^**−1**^	**Selectivity,** φ**, %**	**Treatment with water after separation and cleaning,** ***R*** **, m** ^**−1**^
1	31	5.05 × 10^13^	34	2.53 × 10^13^
2	32	5.15 × 10^13^	35	2.66 × 10^13^
3	32	5.18 × 10^13^	36	2.68 × 10^13^
4	32	5.21 × 10^13^	36	2.69 × 10^13^
5	32	5.23 × 10^13^	36	2.70 × 10^13^
Chemical regeneration				
6	32	5.07 × 10^13^	34	2.56 × 10^13^

However, after the fifth cycle followed by long-time storage in deionized water, colonies of microorganisms were found on the outer surface of the pristine and modified membranes. This is evidently due to adhesion of microorganisms during separation process. Adhesion is possible on the outer surface, since their penetration inside membranes is difficult due to steric factor. In owing to this, the membranes were treated with a HCl solution (see subsection ‘Separation process’). After cleaning, both selectivity of the membranes and their hydrodynamic resistance have been found to be close to those for the first cycle due to the removal of the precipitate from pores.

Thus, the advantage of the modified membrane is its lower resistance evidently due to stability against accumulation of organics inside pores. However, long-time operation requires also protection of the outer surface of the membranes or their regular disinfection. A solution of the problems is outside the scope of this work.

## Conclusions

Modification of polymer track membranes, which was performed by insertion of inorganic filler like ZHP inside their macropores, allows us to obtain the inner active layer in opposite to majority of commercially available membranes. The mechanism of stepwise modification is as follows. First, the macropores of the polymer are blocked with aggregates of nanoparticles a size of which is 10 nm. The ‘corks’ isolate wide cavities and provide permittivity of the membrane towards cations, as shown by measurements of membrane potential. No considerable increase of the modifier amount was found after further modification stages, since secondary porosity limits ZHP deposition inside the polymer.

Both the pristine and composite membranes were tested for baromembrane separation of corn distillery. In the case of modified separator, precipitation occurs directly onto the outer surface in opposite to the pristine membrane, for which deposition inside pores was found. The precipitate can be easily removed from the surface. Location of the active layer inside membrane prevents its mechanical damage.

The directions of further investigations are evidently purposeful regulation of the filler amount inside the membranes, establishment of interrelation between this characteristic and functional properties of the membranes, modification of different types of porous polymers and application of the composites to solution of different tasks of baromembrane separation. Moreover, the protection of outer surface of the membranes against biogenic fouling or regular disinfection is needed to provide their long lifetime, especially in media of liquids of biological origin.

## Abbreviations

*α*: is the parameter related to mass content of the modifier (dimensionless)
*β*: is the parameter related to microporosity
*ε*: porosity (dimensionless)
*ε*_*d*_: related dielectric permittivity (dimensionless)
*ε*_0_: dielectric permittivity of free space (8.85 × 10^−12^ F m^−1^)
*η*: surface charge density (C m^−2^)
*κ*: electric conductivity (Ohm^−1^ m^−1^)
*ρ*_*p*_: particle density (g cm^−3^)
*ρ*_*b*_: bulk density (g cm^−3^)
$\overline{}$: corresponds to membrane
/: corresponds to active layer
*A*: area (m^2^) or ion-exchange capacity (mmol g^−1^)
*a*: activity (mol m^−3^)
An: anion
*C*: concentration (mol m^−3^)
Cat: cation
*d*: particle diameter (m, nm)
EDL: electric double layer
*E*_*m*_: membrane potential (V)
*F*: Faraday constant (96,485 A s mol^−1^)
*J*: flux (dm^3^ m^2^ h^−1^)
*k*: shape coefficient (dimensionless)
*l*: thickness (m)
*m*: mass fraction of the modifier (dimensionless)
Micr: micropores
*P*: pressure (Pa)
*R*: gas constant (8.31 J mol^−1^ K^−1^) or hydrodynamic resistance (m^−1^)
*r*: pore radius (m, nm, A)
*S*: specific surface area (m^2^ g^−1^)
*t*: transport number (dimensionless)
*T*: temperature (K)
*τ*: time, (s, min, h)
u: mobility (m^2^ V^−1^ s^−1^)
V: pore volume (cm^3^ g^−1^) or permeate volume (dm^3^)
z: charge number (dimensionless)
ZHP: zirconium hydrophosphate
